# Deep Learning Application for Vocal Fold Disease Prediction Through Voice Recognition: Preliminary Development Study

**DOI:** 10.2196/25247

**Published:** 2021-06-08

**Authors:** Hao-Chun Hu, Shyue-Yih Chang, Chuen-Heng Wang, Kai-Jun Li, Hsiao-Yun Cho, Yi-Ting Chen, Chang-Jung Lu, Tzu-Pei Tsai, Oscar Kuang-Sheng Lee

**Affiliations:** 1 Institute of Clinical Medicine National Yang Ming Chiao Tung University Taipei Taiwan; 2 Department of Otorhinolaryngology-Head and Neck Surgery Fu Jen Catholic University Hospital Fu Jen Catholic University New Taipei City Taiwan; 3 School of Medicine College of Medicine Fu Jen Catholic University New Taipei City Taiwan; 4 Voice Center Department of Otolaryngology Cheng Hsin General Hospital Taipei Taiwan; 5 Muen Biomedical and Optoelectronic Technologist Inc Taipei Taiwan; 6 Graduate Institute of Business Administration Fu Jen Catholic University New Taipei City Taiwan; 7 Department of Orthopedics China Medical University Hospital Taichung Taiwan; 8 Stem Cell Research Center National Yang Ming Chiao Tung University Taipei Taiwan; 9 Department of Medical Research Taipei Veterans General Hospital Taipei Taiwan

**Keywords:** artificial intelligence, convolutional neural network, dysphonia, pathological voice, vocal fold disease, voice pathology identification

## Abstract

**Background:**

Dysphonia influences the quality of life by interfering with communication. However, a laryngoscopic examination is expensive and not readily accessible in primary care units. Experienced laryngologists are required to achieve an accurate diagnosis.

**Objective:**

This study sought to detect various vocal fold diseases through pathological voice recognition using artificial intelligence.

**Methods:**

We collected 189 normal voice samples and 552 samples of individuals with voice disorders, including vocal atrophy (n=224), unilateral vocal paralysis (n=50), organic vocal fold lesions (n=248), and adductor spasmodic dysphonia (n=30). The 741 samples were divided into 2 sets: 593 samples as the training set and 148 samples as the testing set. A convolutional neural network approach was applied to train the model, and findings were compared with those of human specialists.

**Results:**

The convolutional neural network model achieved a sensitivity of 0.66, a specificity of 0.91, and an overall accuracy of 66.9% for distinguishing normal voice, vocal atrophy, unilateral vocal paralysis, organic vocal fold lesions, and adductor spasmodic dysphonia. Compared with the accuracy of human specialists, the overall accuracy rates were 60.1% and 56.1% for the 2 laryngologists and 51.4% and 43.2% for the 2 general ear, nose, and throat doctors.

**Conclusions:**

Voice alone could be used for common vocal fold disease recognition through a deep learning approach after training with our Mandarin pathological voice database. This approach involving artificial intelligence could be clinically useful for screening general vocal fold disease using the voice. The approach includes a quick survey and a general health examination. It can be applied during telemedicine in areas with primary care units lacking laryngoscopic abilities. It could support physicians when prescreening cases by allowing for invasive examinations to be performed only for cases involving problems with automatic recognition or listening and for professional analyses of other clinical examination results that reveal doubts about the presence of pathologies.

## Introduction

The impact of a voice disorder has been increasingly recognized as a public health concern. Dysphonia influences the quality of physical, social, and occupational aspects of life by interfering with communication [1]. A nationwide insurance claims data analysis of treatment seeking for dysphonia showed a prevalence rate of 0.98% among 55 million individuals [2], and this rate reached 2.5% among those older than 70 years [2]. However, the overall dysphonia incidence for the aging population is estimated to be much higher (12%-35%) [3], which may imply that dysphonia is commonly overlooked by patients, resulting in underdiagnosis.

According to the state-of-the-art clinical practice guidelines for dysphonia of the American Academy of Otolaryngology-Head and Neck Surgery Foundation, a laryngoscopic examination is recommended if dysphonia fails to resolve or improve within 4 weeks [4]. A comparison of diagnoses made by primary care physicians and those made by laryngologists and speech-language pathologists with experience in interpreting stroboscopy at multidisciplinary voice clinics indicated that the primary care physicians’ diagnoses of dysphonia were different in 45%-70% of cases [4]. However, the laryngoscopic examination is an invasive procedure. To achieve an accurate diagnosis, it must be performed by an experienced laryngologist. The examination equipment is expensive and not generally available in primary care units. In places without sufficient medical resources, delayed diagnoses and treatments are common [5]. Therefore, a noninvasive diagnostic tool is needed to resolve this problem. Although this tool cannot replace the laryngoscopic examination by an experienced physician, it is worthwhile to develop because a noninvasive tool to screen significant clinical conditions could encourage patients to visit a voice clinic for further evaluation.

Several recent studies have attempted to distinguish normal and abnormal voices by using various machine learning–based classifiers that have the potential for detecting pathological voices [5-9]. To date, the highest accuracy of pathological voice detection achieved by using a deep neural network has been 99.32% [5]. However, the differential diagnosis of various types of pathological voices has not been widely reported. The vibration patterns of vocal fold observed by high-speed video for common vocal fold diseases, including vocal atrophy, unilateral vocal paralysis, and organic vocal fold lesions, are completely different [10]. We hypothesized that different vibration patterns could result in different voice features. This study sought to detect various vocal fold diseases through pathological voice recognition using a deep learning approach.

## Methods

### Sample Collection

This study was performed following the principles expressed in the Declaration of Helsinki, and approved by the Institutional Ethics and Research Committee of Cheng Hsin General Hospital and Fu Jen Catholic University. Voice samples were obtained from the Voice Center of Chen Hsin General Hospital and the Department of Otorhinolaryngology-Head and Neck Surgery of Fu Jen Catholic University Hospital. These samples included 189 normal voice samples and 552 samples of voice disorders, including vocal atrophy (n=224), unilateral vocal paralysis (n=50), organic vocal fold lesions (n=248), and adductor spasmodic dysphonia (n=30). Voice samples of a sustained vowel sound /a:/ followed by continuous speech of a Mandarin passage [11] (Multimedia Appendix 1) were recorded at a comfortable loudness level with a microphone-to-mouth distance of approximately 15-20 cm using a high-quality microphone with a digital amplifier and a 40- to 45-dB background noise level. The sampling rate was 44,100 Hz with 16-bit resolution, and data were saved in an uncompressed .wav format.

### Comparison and Evaluation

We first divided the 741 samples into 2 sets: 593 samples for the training set and 148 samples for the testing set. Using computer-based randomization, we selected 152 of the 189 normal voice samples, 40 of the 50 unilateral vocal paralysis samples, 24 of the 30 adductor spasmodic dysphonia samples, 179 of the 224 vocal atrophy samples, and 198 of the 248 organic vocal fold lesion samples for the training set (Table 1).

**Table 1:** Details of the voice samples used for experiments (N=741).

Sample	Training set (n=593)	Test set (n=148)
Normal	152	37
Unilateral vocal paralysis	40	10
Adductor spasmodic dysphonia	24	6
Vocal atrophy	179	45
Organic vocal fold lesions	198	50

To manage the limited size of the training set, we used a mix-up approach for data augmentation [12]. The mix-up approach has been applied for audio scene classification using convoluted neural networks (CNNs) to reduce overfitting and obtain higher prediction accuracy [13]. We randomly selected 2 voice files and mixed them into 1 voice file with randomly selected weights to construct the virtual training examples. Next, we randomly cropped each of these voice files to achieve 10 voice files with a length of 11.88 seconds (plateau point of the training length within the graphics processing unit memory limitations of our hardware, according to our preliminary tests). Additionally, we used oversampling to adjust the class distribution of the data [14].

A 2D graph is ideal for extracting features when using CNNs. Therefore, we performed Mel frequency cepstral coefficients (MFCCs) for the processed voice file to obtain a spectrogram. Feature extraction from MFCCs was performed using pre-emphasis, windowing, fast Fourier transform, Mel filtering, nonlinear transformation, and discrete cosine transform [15]. The first feature consisted of 40-dimension MFCCs [16,17]. Next, for the second and third features, we calculated the MFCC trajectories over time (delta MFCCs) and the second-order delta of MFCCs. Therefore, there were 3 channels of input features that could be considered a color image (ie, red–green–blue in the computer vision field).

CNNs have distinct feature representation–related characteristics, among which the lower layers provide general feature-extraction capabilities and the higher layers include information that is increasingly more specific to the original classification task [18]. This allows verbatim reuse of the generalized feature-extraction and representation of the lower CNN layers; the higher layers are fine-tuned toward secondary problem domains with characteristics related to the original. Therefore, instead of designing a new CNN with random parameter initialization, it is more suitable to adopt a pretrained CNN and fine-tune its parameterization toward specific classification domains. Spectrograms were quite different from normal images at first glance. However, the low-level features, including edges, corners, and shapes, were common in the normal images and spectrograms [19]. In a previous study, a spectrogram-based crowd sounds analysis using pretrained CNN models from the ImageNet data set showed great accuracy when distinguishing crowd emotions [19]. Another study also proved that pretrained CNN models yielded better performance than nontrained CNN models for classifying normal or pathological cases [18]. We used different CNN architectures, such as EfficientNet-B0 to B6 [20], SENet154 [21], Se_resnext101_32x4d [21], and se_resnet152 [21] models, from the ImageNet data set that have been pretrained for transfer learning. We classified pathological conditions into 2 (normal voice; adductor spasmodic dysphonia plus organic vocal fold lesions plus unilateral vocal paralysis plus vocal atrophy), 3 (normal voice; adductor spasmodic dysphonia; organic vocal fold lesions plus unilateral vocal paralysis plus vocal atrophy), 4 (normal voice; adductor spasmodic dysphonia; organic vocal fold lesions; unilateral vocal paralysis plus vocal atrophy), or 5 (normal voice; adductor spasmodic dysphonia; organic vocal fold lesions; unilateral vocal paralysis; vocal atrophy) different conditions and trained the CNN. For the final prediction of an input instance, we used the maximum probability to obtain the label.

In terms of hyperparameter settings for fine-tuning among the training set, 474 of 593 samples (79.9%) were used for initial training and 119 of 593 samples (20.1%) were used for validation. We added the dropout function and different data augmentation methods to prevent the model from overfitting in our data set [22,23]. The dropout rate was set at 0.25-0.5 for regularization. Then, we trained the model using minibatches of 32 that were selected based on memory consumption [24]. The learning rate was tuned based on cosine annealing and a 1–cycle policy strategy [25,26]. By using the cosine annealing schedule, the model repeatedly fitted the gradient to the local minimum. The network was trained end-to-end using the Adam optimization algorithm, and it optimized the cross-entropy as a loss function [27]. For different classification problems in the model head, we applied a SoftMax layer as an output layer for multiclass classification or a sigmoid layer for binary classification. Finally, we assembled the model by average output probability to receive more robust results to minimize the bias of prediction error to improve the prediction accuracy of the CNN models [25]. The machine learning process was performed using Python 3.8 (Python Software Foundation) and PyTorch 1.7.1 for Ubuntu 18.04 (Facebook's AI Research lab [FAIR]). Furthermore, we invited 2 laryngologists and 2 general ear, nose, and throat (ENT) physicians who could speak Mandarin to categorize the voice samples of the testing sets into 5 classifications. We compared their classifications with those of our model.

### Statistical Analysis

The effectiveness of our model was evaluated by several metrics, including accuracy, sensitivity, specificity, F1 score, receiver-operating characteristic (ROC) curve, and area under the curve (AUC). All metrics were calculated using Python.

## Results

**Figure 1:**
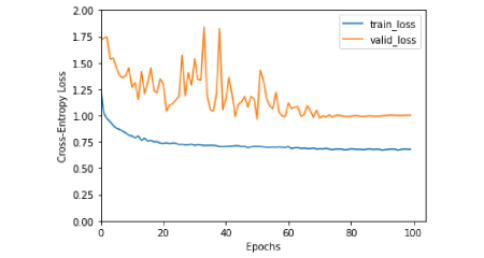
Illustration of the changes of the loss function value over the training and validation sets.

Voice samples in this study were composed of a sustained vowel sound and a continuous essay speech. We first compared the results by training different parts: the vowel sound alone, the essay alone, and the whole voice sample (ie, the vowel sound and essay). Because the vowel sound and essay group (F1 score=0.65) achieved better F1 scores than the vowel sound group (F1 score=0.54) and the essay group (F1 score =0.57), we applied whole voice samples during subsequent machine learning. Figure 1 shows the changes in the loss function value over the training and validation sets, which demonstrated that our model could converge after running the optimization for a number of epochs.

**Figure 2:**
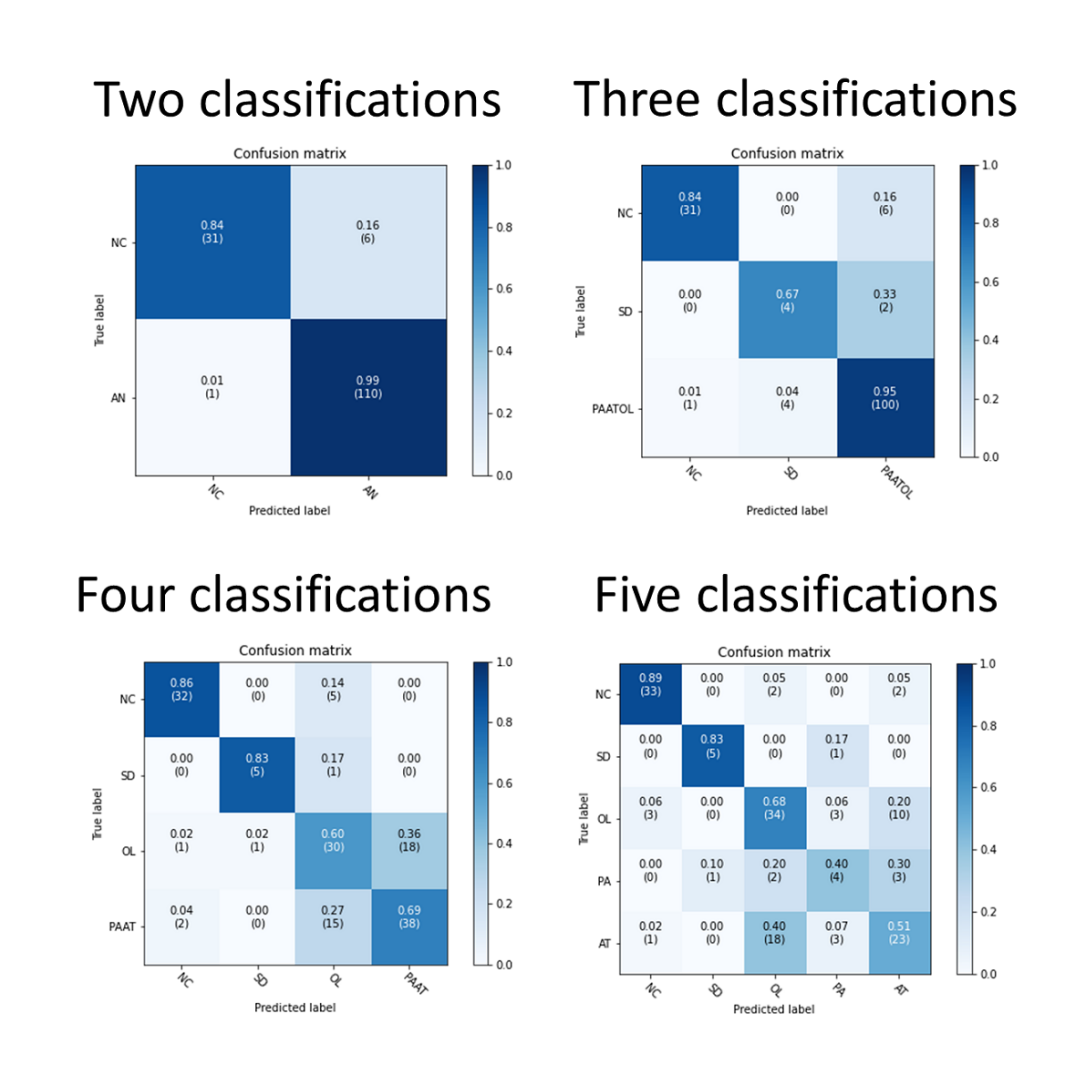
Confusion matrix of 2, 3, 4, and 5 classifications. AN = pathological voice; NC = normal voice; SD = adductor spasmodic dysphonia; PAATOL = unilateral vocal paralysis/vocal atrophy/organic vocal fold lesions; OL = organic vocal fold lesions; PAAT = unilateral vocal paralysis/vocal atrophy; PA = unilateral vocal paralysis; AT = vocal atrophy.

**Figure 3:**
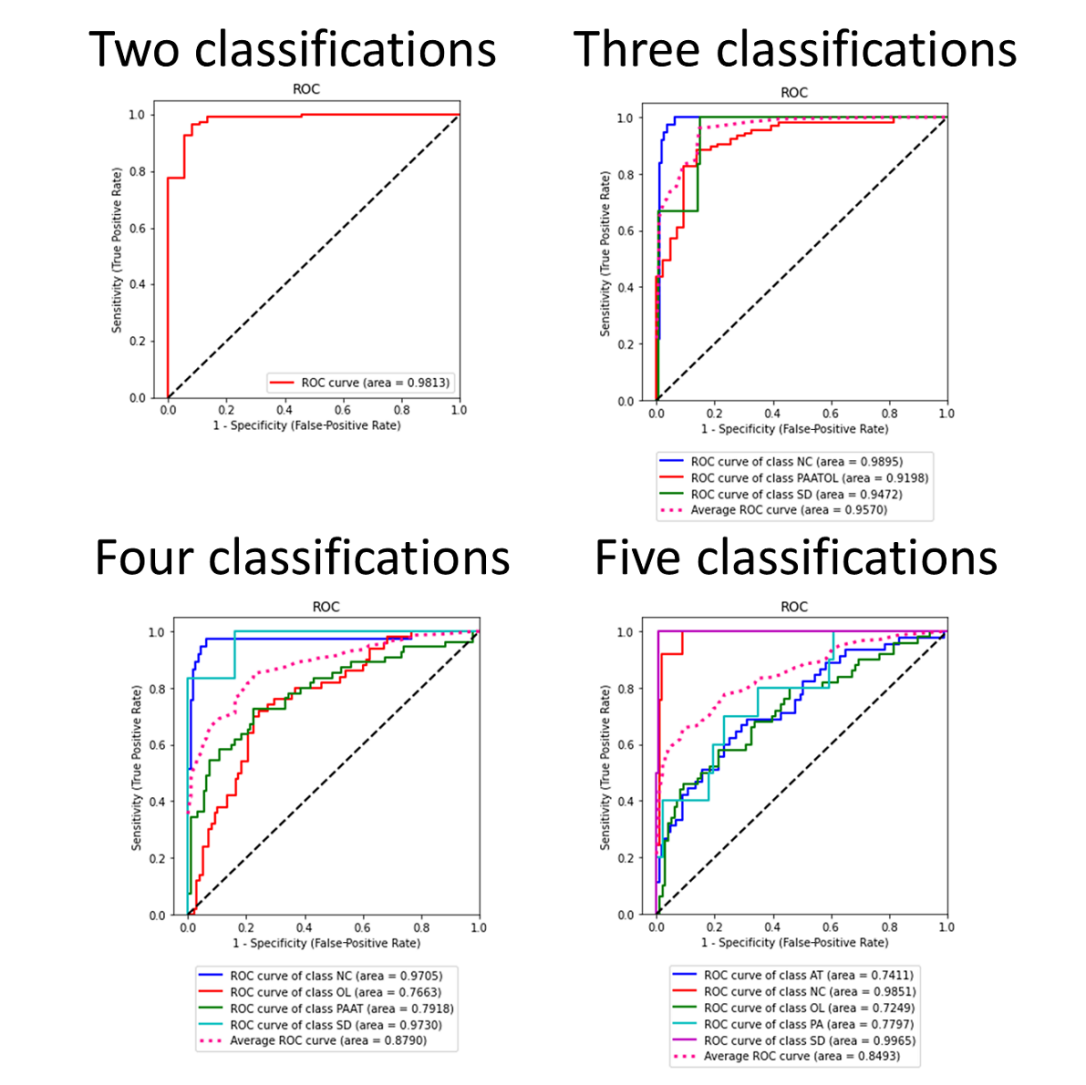
Receiver operating characteristic curves of 2, 3, 4, and 5 classifications. NC = normal voice; SD = adductor spasmodic dysphonia; PAATOL = unilateral vocal paralysis/vocal atrophy/organic vocal fold lesions; OL = organic vocal fold lesions; PAAT = unilateral vocal paralysis/vocal atrophy; PA = unilateral vocal paralysis; AT = vocal atrophy.

Table 2 presents the training results for the different classification methods, including 2 (normal voice; adductor spasmodic dysphonia plus organic vocal fold lesions plus unilateral vocal paralysis plus vocal atrophy), 3 (normal voice; adductor spasmodic dysphonia; organic vocal fold lesions plus unilateral vocal paralysis plus vocal atrophy), 4 (normal voice; adductor spasmodic dysphonia; organic vocal fold lesions; unilateral vocal paralysis; vocal atrophy), or 5 (normal voice; adductor spasmodic dysphonia; organic vocal fold lesions; unilateral vocal paralysis; vocal atrophy) different conditions trained by the CNN. The 2-classification condition could equally distinguish pathological voices from normal voices. In our model, the accuracy of pathological voice detection reached 95.3%; the sensitivity was 99%, specificity was 84%, and AUC was 0.98. Using the 3-classification condition, we aimed to identify adductor spasmodic dysphonia patients from those with other vocal fold pathologies. The accuracy was 91.2%, sensitivity was 82%, specificity was 93%, and AUC was 0.91. Using the 4-classification condition, vocal atrophy and unilateral vocal paralysis could be clinically grouped as “glottic insufficiency.” For this condition, the accuracy was 71.0%, sensitivity was 75%, specificity was 89%, and AUC was 0.88. Using the 5-classification condition, the accuracy was 66.9%, sensitivity was 66%, specificity was 91%, and AUC was 0.85. Figure 2 shows the confusion matrix of these results. Figure 3 shows the ROC curves of these results.

**Table 2:** Performance of the artificial intelligence model for classifying voice disorders under different classification conditions.

Class	Sensitivity	Specificity	Accuracy, %	F1 score	Average area under the curve values
2	0.99	0.84	95.3	0.97	0.98
3	0.82	0.93	91.2	0.80	0.96
4	0.75	0.89	71.0	0.75	0.88
5	0.66	0.91	66.9	0.66	0.85

**Figure 4:**
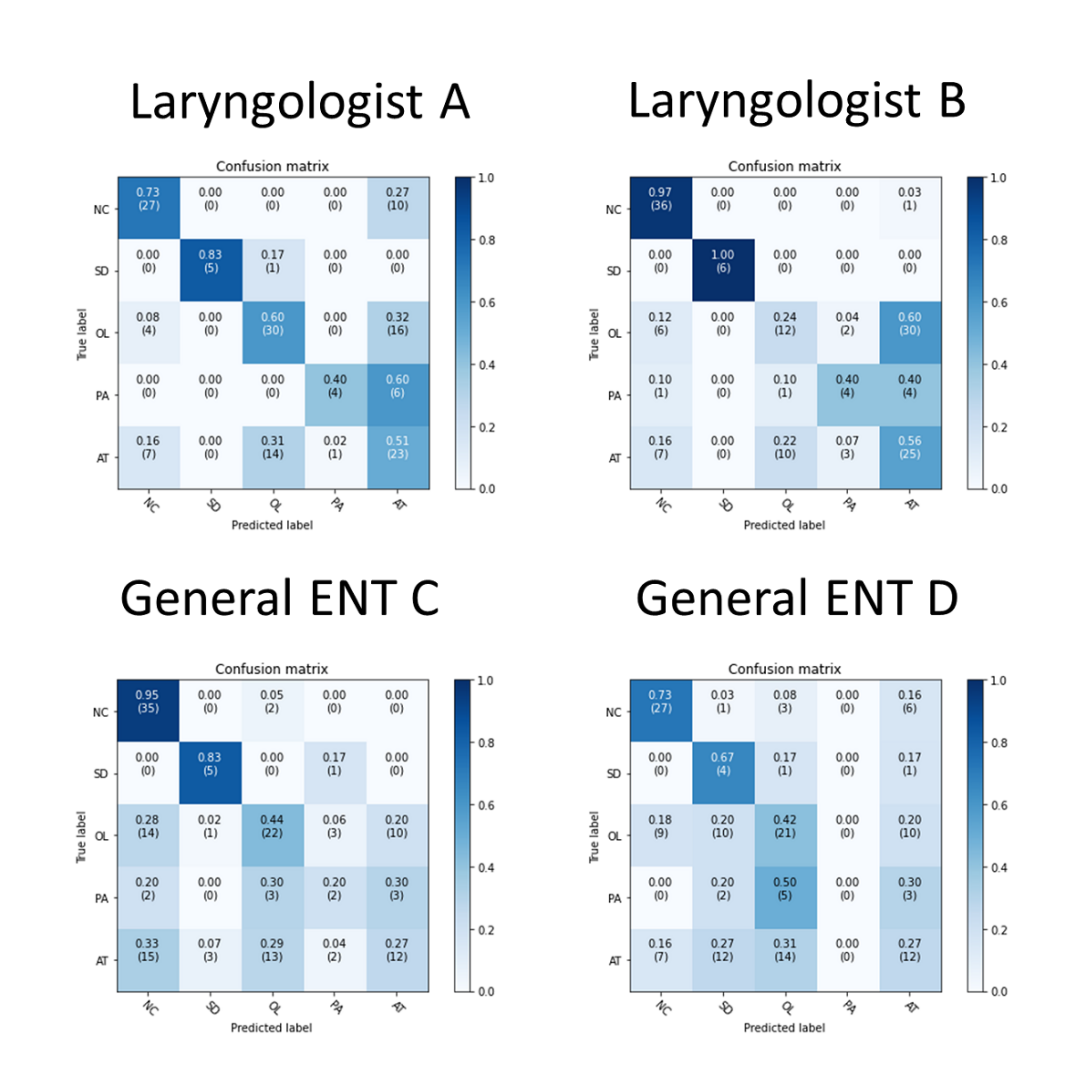
Confusion matrix of 5 classifications in human specialists. NC = normal voice; SD = adductor spasmodic dysphonia; OL = organic vocal fold lesions; PA = unilateral vocal paralysis; AT = vocal atrophy.

Furthermore, we invited four ENT specialists to identify vocal fold pathology by voice using these 5 classifications. The results are shown in Table 3 and Figure 4. The accuracy rates were 60.1% and 56.1% for the 2 laryngologists and 51.4% and 43.2% for the 2 general ENT specialists.

**Table 3:** Comparison of the performance for a 5-classification condition by our artificial intelligence model and 4 human experts.

Test participants	Sensitivity	Specificity	Accuracy, %
Deep learning model	0.66	0.91	66.9
Laryngologist A (11 years of experience)	0.61	0.89	60.1
Laryngologist B (10 years of experience)	0.63	0.88	56.1
General ENT^a^ C (8 years of experience)	0.54	0.88	51.4
General ENT D (14 years of experience)	0.42	0.85	43.2

^a^ENT: ear, nose, and throat.

## Discussion

### Principal Findings

During this study, we built a CNN model that could distinguish, with high specificity (91%), different pathological voices attributable to common vocal diseases based on voice alone. To the best of our knowledge, no previous study has used artificial intelligence to distinguish different types of pathological voices speaking Mandarin. Using our model, we obtained better results by training the CNN with a whole voice sample than by training it with the vowel sound only or with the essay speech only.

Our model could distinguish normal voice and adductor spasmodic dysphonia with great performance for the 5-classification condition (AUC values: 0.985 and 0.997, respectively). The overall accuracy of our model was also better than that of all ENT specialists participating in the study. This was compatible with our clinical observation that the first impression of the pathological voice is usually over-ruled by the laryngoscopic examination. Additionally, laryngologists demonstrated higher accuracy when diagnosing voice disorders than general ENT specialists. This may imply that it would be possible to improve the accuracy of human physicians in terms of their impressions of pathological voices by increasing clinical experience. After comparing the accuracy of each classification, we found that artificial intelligence was markedly better than laryngologists when identifying organic vocal fold lesions (artificial intelligence, 68%; laryngologist A, 60%; laryngologist B, 24%). However, laryngologists were slightly better at vocal atrophy identification (artificial intelligence, 51%; laryngologist A, 51%; laryngologist B, 56%).

Organic vocal fold lesions, unilateral vocal paralysis, and vocal atrophy could result in a closure gap during phonation, inducing a weak and breathy sound [28-30], and vocal fold tension imbalance, inducing diplophonia (when a voice is perceived as being produced with 2 concurrent pitches) [31]. Specifically, in the case of organic vocal fold lesions during vibration, the lesion divided the fold into 2 oscillators. However, in the case of unilateral vocal paralysis, vibrating frequencies were different between the normal vocal fold and paralysis vocal fold. Vocal atrophy will show a breakdown of vibration with a visible repetition in the loss of normal vibration every few glottal cycles [10]. However, the difference in the vibration pattern could only be observed by high-speed video and multislice digital videokymography [10], and the resulting pathological voice is difficult for humans to identify. We speculated that our model may identify related features through deep learning to achieve better outcomes.

Laryngologists could distinguish aged and young patients, and they could validate their judgment during the test based on their knowledge. Vocal atrophy is the most common vocal fold pathology in older patients [3]. Therefore, laryngologists may classify the pathological voice as vocal atrophy if they judged that the voice was that of an aged person.

Regarding misclassification, we have found that our model could successfully identify normal voice and spasmodic dysphonia. However, it was relatively difficult to differentiate organic vocal lesions, unilateral vocal paralysis, and vocal atrophy from each other. Although the vibration patterns were different for these 3 diseases, the different severity levels of disease could result in different degrees of hoarseness. For example, with tiny vocal nodules compared with huge vocal polyps, unilateral vocal paralysis with fair compensation compared with unilateral vocal paralysis with a huge closure gap, and vocal atrophy with a mild anterior closure gap compared with vocal atrophy with a huge closure gap, there could be different degrees of hoarseness in the same group. We assumed that the less severe cases in each group may not show the typical pathological vibration pattern. Further studies are needed to validate our hypothesis.

Four human specialists required 40-80 minutes to identify 148 voice samples of the test set; however, our model only required 30 seconds to perform the same task. The processing time of our model is quite promising in terms of the development of future screening tools.

### Comparison With Prior Work

Most previous studies have used sustained vowel sounds for pathological voice detection [5,7,8]. However, other studies have used continuous speech samples for analyses [6]. CNNs extract features automatically from the spectrogram of voice recordings for dysphonia diagnosis, and a larger amount of training data yields better results [32]. Therefore, the CNN used here may have extracted more features from these entire voice samples, thereby achieving better training results with our model.

In this study, we used our voice database for the deep learning approach. The most widely used voice disorder database is the Massachusetts Eye & Ear Infirmary (MEEI) Voice Disorders Database (commercially available from KayPENTAX Inc.). The MEEI voice samples (53 normal and 662 pathological voices) are composed of the vowel /ah/ (53 normal and 657 pathological voices) and the utterance of a sentence (“When the sunlight strikes raindrops in the air, they act as a prism and form a rainbow”) [33]. However, the voice recordings in the MEEI database were recorded at various sampling rates (10, 25, and 50 kHz), and normal and pathological voice recordings were recorded in 2 different environments [32]. Therefore, it was not clear whether artificial intelligence was classifying voice features or environments when trained using the MEEI samples.

The other widely used voice disorder database is the Saarbruecken Voice Database, which contains voice recordings from more than 2000 individuals. Each participant file contains recordings of sustained vowel sounds of /a/, /i/, and /u/ in low, neutral, high, and low–high–low pitches, as well as a continuous speech sentence (“Guten Morgen, wie geht as Ihnen?”). All these samples were recorded at 50-kHz sampling rates and 16-bit resolution [32]. The Saarbruecken Voice Database is considered to be superior to the MEEI database because it uses the same recording environment and the same sampling rates. However, it contains 71 different dysphonia pathologies and many patients recorded in this database had multiple disorders. Therefore, it is difficult to achieve denotation before machine learning.

Our database has several advantages. First, all voice data were from patients visiting our clinics who had detailed chart documents that were carefully reviewed by 2 experienced laryngologists (H-CH and S-YC). Therefore, the quality of the primary data was better than that of the primary data of other studies during which voice data were retrieved from a public database. Second, all voice data were recorded using 44.1-kHz sampling rates and 16-bit resolution, which comprise the standard audio CD format. This widely used format could increase the usability of this data set. Third, we focused on 4 vocal fold diseases that were chosen by experienced laryngologists based on the cause of hoarseness, clinical significance, and prevalence of the disease.

In terms of the cause of hoarseness, adductor spasmodic dysphonia is a focal laryngeal dystonia characterized by irregular and uncontrolled voice breaks that interrupt normal speech [34]. However, organic vocal fold lesions, unilateral vocal paralysis, and vocal atrophy could induce a breathy sound with a different diplophonia pattern [10]. The voice pattern of adductor spasmodic dysphonia is quite different from that of the other pathologies included in this study, and classic cases of adductor spasmodic dysphonia could be diagnosed based on voice alone by experienced laryngologists. The accuracy rates of adductor spasmodic dysphonia among laryngologists using the 5-classification condition were 100% and 83% during this study. Therefore, we anticipated that the sensitivity and specificity for diagnosing adductor spasmodic dysphonia could be higher than those of other categories. However, during the first attempt at CNN training, the accuracy of adductor spasmodic dysphonia identification was poor (data not shown). When we attempted to splice the original voice file into 1-second clips while training the model, we found that the voice break in adductor spasmodic dysphonia did not always emerge within every 1-second period. After prolonging the duration of the voice clips in the training model, the results improved substantially. This also emphasized that the domain knowledge could significantly influence the training results by tuning the training model according to real clinical conditions.

According to a meta-analysis, in terms of clinical significance, patients with neurologic voice disorders have more challenges than patients with inflammatory or traumatic laryngeal diseases [1]. Specifically, adductor spasmodic dysphonia showed the worst Voice Handicap Index (VHI) score, followed by unilateral vocal paralysis [1]. This result was compatible with our clinical observation that adductor spasmodic dysphonia could markedly interfere with communication and socialization during the daily lives of patients. Although adductor spasmodic dysphonia is a rare disease with a prevalence of 14 out of 100,000 [35], it is worthwhile to offer a model for rapid screening because the symptoms can be treated easily and effectively by regular intralaryngeal botulinum toxin type A injections or surgery [35].

According to VHI scores, unilateral vocal paralysis could also induce a severe voice handicap [1]. The most common cause of unilateral vocal paralysis is an idiopathic or postviral infection, which accounts for 67% of cases [36]. However, 6% of patients have underlying malignancies that invade the recurrent laryngeal nerve or vagus nerve [36]. Computed tomography of the skull base, neck, and chest is often recommended during the search for a potential cause of the voice disorder [37]. Thyroid disease, including benign nodules, thyroid malignancy, thyroiditis, hyperthyroidism, and hypothyroidism, may also result in vocal fold paresis [38]. Heman-Ackah et al [38] reported that 47.4% of patients with unilateral vocal paralysis are diagnosed with concurrent thyroid disease. Therefore, it is important to determine an early diagnosis of unilateral vocal paralysis to investigate the existence of underlying disease.

Organic vocal fold lesions comprise benign lesions, such as nodules, polyps, cysts, polypoid vocal folds, precancerous leukoplakia, and malignant lesions [39,40]. The cause of hoarseness with benign and malignant vocal fold lesions involves changes in the laryngeal mucosa and mass effects [10,40]. To date, it has been difficult to differentiate organic vocal fold lesions further by voice alone because they involve various pathologies. However, it is worthwhile to inform patients about the possibility of organic vocal fold lesions and to advise them to undergo further investigations. Early stage malignant lesions and benign lesions could be treated with office-based surgery, which is safer and relatively inexpensive compared with surgery in the operating room [29,41,42]

The most common cause of vocal atrophy is aging. Aging may result in atrophic musculature and a thinner lamina propria of the vocal fold [43]. However, vocal atrophy can also occur in a relatively young population [28]. It may result from a congenital anomaly or prolonged laryngopharyngeal reflux [44]. The symptoms associated with vocal atrophy are relatively subtle compared with those of other vocal fold diseases [45]. The concern about significant underlying diseases is also reduced with vocal atrophy. However, vocal atrophy is the most common vocal fold pathology among patients older than 65 years [3]. With the aging of the population, vocal atrophy may become a significant geriatric issue in the future.

### Limitations

Our study had some drawbacks. First, all the voice files were recorded in the studio with a silent environment, with sensitive audio-recording technology, and using a certain format. Further studies are needed to validate this approach in different recording environments. Second, all the voice data in this study are from Mandarin speakers. Further studies are needed to compare the results of speakers of different languages. Third, the voice sample numbers of each class were unequal because of the different disease prevalence. We have applied several data augmentation methods to ameliorate the influence of these unequal data. Fraile et al [46] showed that laryngeal pathology detection using voice records based on MFCC and prior differentiation by sex can significantly improve the performance. Fang et al [47] also showed that a deep neural network combining supervectors with medical records could improve pathological voice classifications. Therefore, in the future, we will combine demographic data with voice records to improve our model.

### Conclusions

We demonstrated that voice alone could be used for common vocal fold disease recognition using a deep learning application after training with our Mandarin pathological voice database. Specifically, adductor spasmodic dysphonia, organic vocal fold lesions, unilateral vocal paralysis, and vocal atrophy could be recognized, which could increase the potential of this approach to be more beneficial than simply distinguishing a pathological voice from a normal voice. This approach shows clinical potential for use during general screening of different vocal fold diseases based on voice and could be included in quick evaluations during general health examinations. It could also be used for telemedicine in remote regions that lack laryngoscopy services in primary care units. Overall, it could support physicians during prescreening of cases by allowing for invasive examinations to be performed only for cases involving problems with automatic recognition or listening and for professional analyses of other clinical examination results that reveal doubts about the presence of pathologies.

## Appendix

Multimedia Appendix 1 Mandarin passage.

## Abbreviations

AUC: area under the curve
CNNs: convoluted neural networks
ENT: ear, nose, and throat
MEEI: Massachusetts Eye & Ear Infirmary
MFCCs: Mel frequency cepstral coefficients
ROC: receiver-operating characteristic
VHI: Voice Handicap Index
