# Supplementary material for: Deep Learning Application for Vocal Fold Disease Prediction Through Voice Recognition: Preliminary Development Study
Source: J Med Internet Res. 2021 Jun 8;23(6):e25247. doi: 10.2196/25247 (PMC8241431; doi:10.2196/25247)
Supplement: Multimedia Appendix 1 [file jmir_v23i6e25247_app1.docx]

我聽到有人敲門，懶懶的說了一聲：「請進來」。門開了，我看見一個年輕人，瘦長的身體，明亮的眼睛，還有一張誠懇的臉，看他臉上的表情以及嚴肅的態度，真像有什麼事情要我幫忙。
